# The Ergonomic Behaviors Evaluation Tool (EBET) based on social cognitive theory for the assembly line workers: development and psychometric assessment

**DOI:** 10.1186/s12889-024-18738-w

**Published:** 2024-05-06

**Authors:** Zakieh Sadat Hosseini, Sedigheh Sadat Tavafian, Omran Ahmadi, Reza Maghbouli

**Affiliations:** 1 Department of Health Education and Health Promotion, Faculty of Medical Sciences, Tarbiat Modares University, Tehran, Iran; 2 Department of Occupational Health Engineering, Faculty of Medical Sciences, Tarbiat Modares University, Tehran, Iran; 3 School of Medicine, Hasheminejad Hospital, Iran University of Medical Sciences, Tehran, Iran

**Keywords:** Ergonomic behaviors, Psychometrics, Assembly line workers, Social cognitive theory

## Abstract

**Background:**

Ergonomic behaviors play a crucial role in preventing work-related musculoskeletal disorders (WMSDs). To measure these behaviors, this research aimed to develop and evaluate an ergonomic behaviors tool (EBET) based on the Social Cognitive Theory (SCT) among women workers on assembly lines (WwAL).

**Methods:**

The study was conducted from December 2022 to January 2023 with a focus on the psychometric assessment of EBET. Initially, a literature review and interviews were carried out to identify crucial concepts and primary items. The questionnaire’s validity was evaluated using the Content Validity Ratio (CVR) and the Content Validity Index (CVI). To determine the domains of the tool, construct validity was examined by administering the items to 270 eligible women. The reliability of the tool was assessed using McDonald’s Omega coefficient.

**Results:**

From a total of 67 primary items, 50 were confirmed. The study demonstrated good validity with CVR = 0.92 and CVI = 0.97, along with reliable results indicated by McDonald’s Omega coefficient of 0.74. The exploratory factor analysis (EFA) revealed ten distinct dimensions: outcome expectations, outcome expectancies, normative beliefs, perceived barriers, social support, observational learning, reinforcement, behavioral skills, self-efficacy, and intention. Together, these dimensions accounted for 66.25% of the variance in the data. Additionally, the confirmatory factor analysis results supported the presence of these ten constructs and demonstrated a satisfactory fit.

**Conclusions:**

EBET is a dependable and valid instrument for evaluating the ergonomic behaviors of workers, utilizing the principles of SCT. Researchers can employ EBET to gather data and implement suitable training interventions to enhance ergonomic behavior among WwAL. However, it is crucial to recognize that EBET may not encompass all facets of ergonomic behaviors. Therefore, it is imperative for future research to prioritize the evaluation of EBET’s suitability among diverse worker populations and to consider additional dimensions of ergonomics to ensure its wider applicability and effectiveness.

## Background

Work-related musculoskeletal disorders (WMSDs) present a considerable occupational health challenge caused by factors such as repetitive movements, poor posture, and prolonged sitting, which can lead to severe pain, restricted mobility, and long-term disability [1, 2]. Occupations involving repetitive motions, like assembly line work and computer-based tasks, require particular consideration as they are more susceptible to causing musculoskeletal disorders (MSDs) [3–6]. WMSDs affect 1.71 billion people worldwide and caused 149 million years of disability in 2019 [7]. Iranian workers have a higher prevalence of WMSDs in upper and lower limbs compared to other countries [8]. As a result of this trend, there has been an increased adoption of educational interventions targeting ergonomic behavior in workplace settings, thereby the heightened significance of measuring ergonomic behavior as a main outcome for evaluating the effectiveness of these interventions [9–11].

The etiology of WMSDs is intricate, influenced by diverse factors such as biomechanical, organizational, psychosocial, and individual risks. These factors, whether direct or indirect, significantly impact musculoskeletal symptoms, rendering identification of a singular cause challenging [12, 13]. Research emphasizes the importance of an ergonomic workplace in preventing MSDs and acknowledges the influence of organizational, environmental, and individual factors on ergonomic practices [14, 15]. Evidence from studies indicates that adopting ergonomic behaviors, such as integrating stretching routines and maintaining proper posture during work, effectively reduces the risk of MSDs in the workplace [16–18].

Social cognitive theory (SCT) is an educational approach that considers individual, environmental, and cognitive components when addressing WMSDs [19, 20]. SCT explores how individuals learn and develop through their interactions with the environment, social interactions, and their cognitive processes [21]. Concepts include observational learning, self-efficacy, the reciprocal relationship between individuals and their environment, cognitive processes and self-regulation, and diverse applications [17, 22, 23]. As a result, SCT is widely regarded as an effective approach for promoting health interventions.

The assessment and validation of tools and methods are crucial for determining the effectiveness and successful integration of new technologies, programs, and approaches in education [24]. These evaluations also help measure the impact and level of success achieved through their implementation [25]. The review of the literature indicates that tools that evaluate MSDs based on educational theories have predominantly been utilized in office settings [2, 11, 26], while the application of such theory-based tools in industrial environments is limited [10, 15, 23]. To the best of our understanding, there is currently no tool available that has been developed for the purpose of measuring ergonomic behaviors on assembly lines, based on SCT. Therefore, with the aim of developing and evaluating the psychometric properties of an instrument based on SCT, this study was conducted. Specifically, the study aimed to develop a valid instrument to assess ergonomic behaviors in Iran.

## Methods

### Development of the questionnaire

#### Theoretical framework

The development of EBET item is based on a conceptual framework base on SCT, which includes cognitive, individual, and environmental dimensions [27]. SCT emphasizes the importance of individual, environmental, and behavioral factors in shaping health behavior. It incorporates key concepts such as self-efficacy, outcome expectations, observational learning, and behavioral capability [28]. By assessing changes in these key factors, SCT allows for the design of interventions and measurement of effectiveness in health behavior change programs [19]. SCT offers a valuable framework for understanding how individuals acquire and adopt new health behaviors. To effectively promote ergonomic behavior among WwAL, it is essential to develop an instrument that specifically focuses on the key constructs within this theory.

#### Generating items through literature review and interview methodology

Both deductive and inductive approaches were employed in crafting the instrument questions for this study. Due to the absence of suitable instruments aligned with our research objectives, two distinct sets of studies were utilized to establish the initial structure of questions pertaining to SCT. Initially, studies grounded in SCT, with a focus on MSDs, underwent review [20, 29]. Additionally, a literature search, albeit not strictly systematic, was conducted using keywords such as ‘worker’, ‘musculoskeletal disorders’, and ‘women’. The aim of this search was to address fundamental inquiries regarding the dimensions of SCT relevant to working women. These inquiries encompassed exploring consequences of ergonomic behaviors, effective sources of approval or attention for such behaviors, major workplace behavior barriers for women, necessary support for ergonomic practices in the workplace, measures including observational learning, and effective workplace incentives. To encourage ergonomic behavior among women, essential behavioral skills required and self-efficacy indicators were also explored. These questions were also incorporated into the interview section. To fortify the questionnaire items and enhance their alignment with the cultural, social, and economic contexts of women working in the industry, qualitative interviews were conducted with a sample of 20 women. Each participant responded to open questions corresponding to those identified in the literature review phase. Interviews lasted between 20 and 45 min, either in person (13 individuals) or by telephone (7 individuals), with all respondents interviewed individually. Verbatim transcriptions of all interviews were manually analyzed using a deductive approach to identify recurring codes corresponding to SCT constructs. The first author led the analysis, subsequently discussing the findings with the other authors. Thematic analysis was employed to extract concepts and insights consistent with existing theory. This analysis guided the creation of additional theory-aligned items and facilitated participant insight. Concepts obtained from the literature review and interviews underwent analysis by the research team, culminating in the preparation of an initial questionnaire containing 67 items. This questionnaire served as the foundation for subsequent psychometric evaluation of the instrument. Table 1 presents the related citations and the examples of interviews.

**Table 1:** Description of the constructs and the related citations and examples from interviews

Construct & Items	Literature Review	Quotes from the Interviews
**Outcome Expectations** -Pain relief -Increase physical ability -Reduce fatigue	Bao et al. (2020) [4], Arghami et al. (2016) [6], Moazzami et al. (2016) [18].	“If I observe my body position while working, my musculoskeletal pain will probably decrease” “Stretching will delay my physical disability”
**Normative Beliefs** -Approval and attention of the team leader and industry expert	Bao et al. (2020) [4], Aje et al. (2018) [23],	“In my workplace, the authorities do not approve such behavior”
**Perceived barriers** -High work pressure -Decreased work speed -Ridicule by colleagues -Lack of ergonomic equipment -Feel embarrassed -Not enough time	Moazzami et al. (2016) [18], Bao et al. (2020) [4], Khalili et al. (2018) [11], Denadai et al. (2021) [10], and Buruck et al. (2019) [30].	“Due to the high amount of work and the short time, it is difficult to perform stretching exercises” “I feel embarrassed to stretch at work” “My colleagues may scold me while doing stretching exercises”
**Social support** -Colleague support -Team leader support -Health and safety expert support -Installation of educational posters -Access to training courses	Bao et al. (2020) [4], Susihono et al. (2021) [15], Aje et al. (2018) [23], Buruck et al. (2019) [30], Villotti et al. (2018) [31], and Henry et al. (2019) [32].	“Here, friends do not invite each other to do stretching exercises or observe the correct body position during work” “If there was an educational poster in my work environment, it would help me more”
**Observational learning** Trying to pay attention, focus, repeat and practice ergonomic behavior	Kwon et al. (2022) [22].	“I usually don’t pay attention to how my colleagues sit correctly” “If my colleagues do stretching exercises, I would definitely do the same.”
**Behavioral skills** Having the skills: -Maintaining body posture while working -Doing a variety of stretching exercises	Muyor et al. (2012) [17], Aje et al. (2018) [23], and Moazzami et al. (2016) [18].	“I don't know how to do stretching exercises in the workplace” “If I knew how to maintain the correct posture of the body, I would do this.”
**Self-efficacy** How easy or difficult: -Doing stretching exercises -Maintaining correct posture while working -Planning to perform ergonomic behavior	Sanaeinasab et al. (2018) [2], Khalili et al. (2018) [11], Denadai et al. (2021) [10], and Moazzami et al. (2016) [18].	“It is very difficult for me to maintain my body position during work” “Stretching is not difficult for me”
**Knowledge** -General & specific musculoskeletal disorder knowledge	Kwon et al. (2022) [22], Akbari, Akbari-Chehrehbargh et al. (2020) [20], and Moazzami et al. (2016) [18].	“I think repetitive work at high speed on an assembly line causes musculoskeletal pain”. “I don’t think low light in the workplace has any effect on causing musculoskeletal pain”
**Reinforcement** Encouragement by: -Team Supervisor -Health and safety expert -Family members	Bao et al. (2020) [4] and Moazzami et al. (2016) [18].	“Every time I stretch at work, the health and safety expert encourages me”

### Psychometrics characteristics of the questionnaire

In order to evaluate the questionnaire's reliability and validity, a variety of measures were utilized. These measures included both quantitative and qualitative assessments of face validity, content validity, construct validity, and reliability.

### Face validity

The assessment of apparent effectiveness is related to the degree to which a measurement tool appears to effectively assess the specific construct it aims to measure [28]. The evaluation involved the integration of qualitative and quantitative approaches. Qualitatively, feedback was obtained from 30 women similar to the target group, who rated the items based on comprehension difficulty, generality, and ambiguity. Quantitatively, item impact scores were calculated. Participants utilized a 5-point Likert scale to rate the importance of each item. The item's impact score was subsequently determined by multiplying the frequency percentage by the corresponding importance rating. Items with an impact score exceeding 1.5 were considered suitable and retained for the subsequent phases of the study.

### Content validity

Content validity plays a crucial role in the development of an instrument by ensuring that the measurement effectively encompasses all elements of a construct [33]. The EBET questionnaire was evaluated using a combination of qualitative and quantitative approaches. During the qualitative evaluation, a group of ten experts examined the items for grammatical accuracy, word usage, and proper placement. Their written comments were considered by the research team. Quantitatively, the expert panel assessed each statement for necessity, usefulness, non-necessity, and non-essentiality. Items with a content validity ratio (CVR) of 0.62 or higher, determined through expert consensus and the Lawshe table, were retained. To determine CVR, the process involved subtracting half of the total number of experts engaged in the evaluation from the number of experts who considered the option 'essential.' The resulting value was then divided by half of the total number of experts participating in the evaluation. Using a 4-point Likert scale, the items were evaluated in terms of simplicity, relevance, and clarity as part of the content validity index (CVI) evaluation. Adequate content validity is achieved when the CVI value is 0.78 or higher [28].

### Evaluation of the questionnaire’s construct validity

A questionnaire designed to assess knowledge levels typically prioritizes confirming its content validity, while the evaluation of its construct validity is often not recommended [34]. As a result, the knowledge questions in our study were not subjected to a construct validity assessment. The appropriate sample size for conducting factor analysis varies among researchers. Some studies suggest that a minimum of 200 participants is sufficient for most cases [35, 36]. Plichta and colleagues propose that having 3–10 participants per variable, or a total of 100–200 respondents, is adequate [37]. Thus, a minimum sample size of 5 times the number of variables was considered, resulting in 270 participants for this stage of our study, given the presence of 48 items. This sample size was used for both exploratory factor analysis (EFA) and confirmatory factor analysis (CFA).

Construct validity is concerned with the extent to which a measurement or assessment instrument effectively captures and measures the intended theoretical construct or concept [21]. The assessment of the EBET’s construct validity involved employing both EFAand CFA methodologies.

### Study design and participants

This study aimed to develop and evaluate a reliable psychometric tool based on SCT to evaluate the ergonomic behaviors of WwAL in Iran. In order to perform EFA, a cross-sectional design was used, and 270 WwAL participated by completing a self-report questionnaire. Random sampling was employed within clusters formed based on industries with WwAL. Inclusion criteria encompass able in reading and writing in Persian, being over 20 years old, and working in an assembly line. Participation in the study was entirely voluntary and confidential, granting participants the freedom to withdraw from the study at their discretion. The survey was administered in a paper-and-pen format, and the purpose of the study and its relevance in preventing MSDs were explained to participants.

### Exploratory factor analysis

The data collection for this study involved utilizing the EBET questionnaire, which underwent assessment for both face and content validity. Principal components analysis with varimax rotation was performed in SPSS version 21 to conduct EFA. The dataset’s appropriateness for factor analysis was assessed by analyzing the Kaiser–Meyer–Olkin (KMO) index and conducting Bartlett's test of sphericity [20]. In this study, the identification of factors and dimensions within the tool was guided by the retention of factors with eigenvalues exceeding one, a widely accepted criterion in factor analysis [33]. Additionally, a scree plot was employed to aid in this selection process.

### Confirmatory factor analysis

The study employed CFA using AMOS 24 software to test the multidimensional hypothesis of EBET. The researchers assessed model fit using several indices, including χ2, which measures the difference between observed data and the proposed model. The examination involved analyzing the ratio of χ2 to degrees of freedom (χ2 / df), with a value close to 1 or less than 3 indicating a good fit. The researchers also considered other fit indices such as comparative fit index (CFI), goodness of fit index (GFI), and normed fit index (NFI), with values above 0.9 indicating good fit. The evaluation also included assessing the root mean square error of approximation (RMSEA), with values below 0.05 indicating excellent fit and values up to 0.08 being deemed acceptable [28].

### Assessment of reliability

**Fig. 1:**
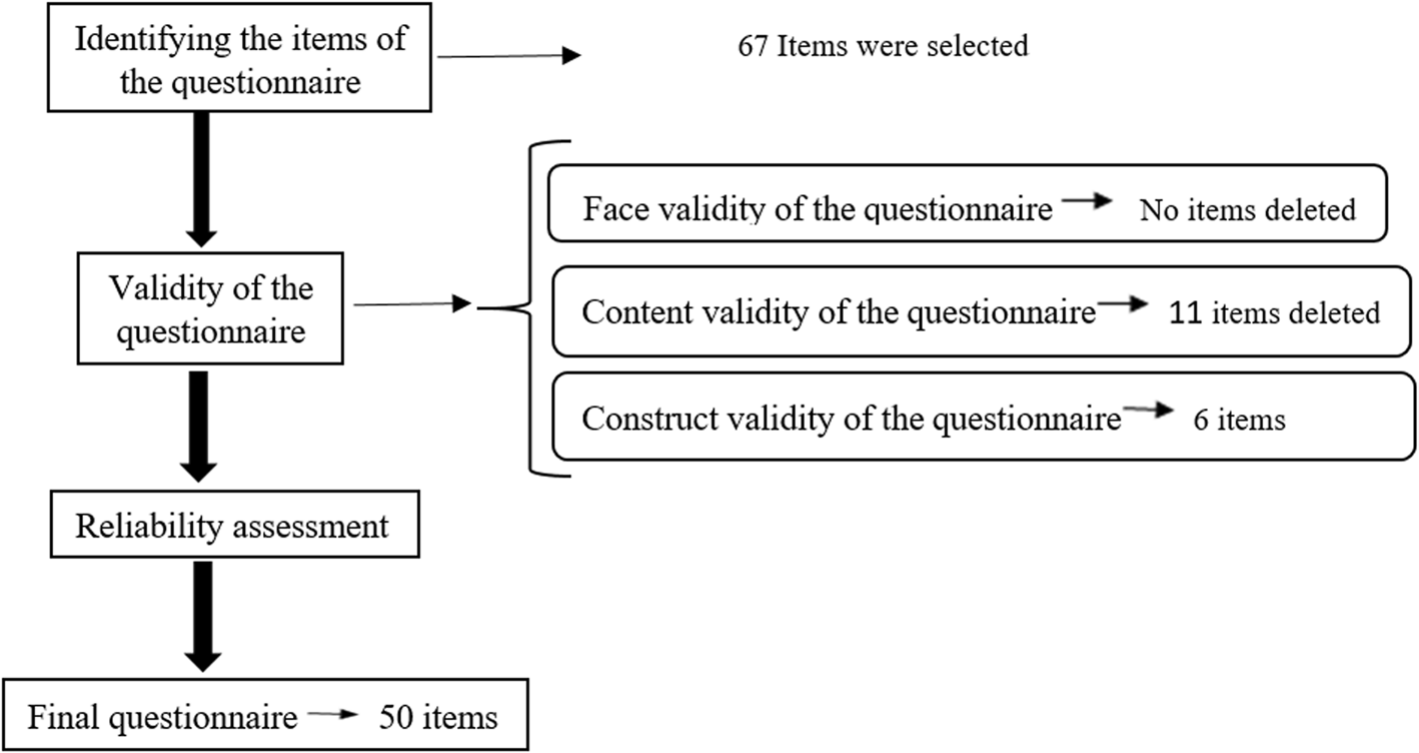
An overview of the procedures involved in designing and evaluating the psychometric properties of EBET

The assessment of internal consistency reliability involved utilizing McDonald’s Omega, a commonly used measure to evaluate reliability. A minimum McDonald's Omega value of 0.70 or greater was established as the acceptable threshold [38]. Figure 1 provides an overview of the steps involved in the design and evaluation of EBET’s psychometric properties.

## Results

### Instrument

This questionnaire comprises two sections: A) Demographic questions including age, height, weight, educational status, marital status, and work experience, and B) Main questions related to EBET. The objective of B section is to assess ergonomic behaviors based on SCT concepts. It includes: outcome expectations (4 items), outcome expectancies (4 items), normative beliefs (3 items), perceived barriers (7 items), social support (5 items), observational learning (4 items), reinforcement (3 items), behavioral skills (4 items), self-efficacy (4 items), and intention (4 items). Respondents provide their opinions using a 5-point Likert scale that encompasses responses ranging from “Strongly Agree” (5) to “Strongly Disagree” (1). Additionally, the questionnaire incorporates eight knowledge questions, facilitating a comprehensive assessment of core structures within SCT.

### Face validity assessment

During the qualitative face validity assessment, certain items were evaluated based on participants’ suggestions. For example, the item “Non-same-sex Colleague” was revised to “Sir”. Furthermore, clarifications were provided in parentheses for terms like “Prolonged Sitting” to ensure a clear understanding of the item. Additionally, certain verbs and items were simplified as part of the review process. During the evaluation of quantitative face validity, all phrases had an impact score exceeding 1.5, resulting in the retention of all items without any removal in this phase.

### Content validity assessment

The content of the items in qualitative CVA was determined based on the recommendations provided by the panel of experts. In the quantitative CVA, a total of 11 items were eliminated as they did not meet the predefined criteria of CVI and CVR. The average score for CVR was 0.92, with a range of 0.85 to 1. Additionally, the average score for CVI was 0.97, ranging from 0.95 to 1. Finally, 56 items remained to be assessed. The whole process of psychometric of EBET and the related changes in the questionnaire are shown in Fig. 1.

### Results of the exploratory factor analysis

The questionnaire was applied to a total of 270 WwAL, The women had an average age of 35.21 ± 7.89 years, with a marriage rate of 58.1%. Table 2 contains further information on demographic factors.

**Table 2:** Demographic characteristics of participants (*n* = 270)

**Variable**		**Frequency** **Mean ± SD**	
Age (year)		35.21 ± 7.89	
work experience (year)		8.19 ± 5.88	
		**Number**	**Percent**
Education	Elementary school	122	45.2
	High school	109	40.4
	Academic	39	14.4
Marital status	Single	61	22.6
	Married	157	58.1
	Widowed	4	1.5
	Divorced	48	17.8
	Very well	4	1.5
Finances	Well	9	3.3
	Moderate	131	48.5
	Poor	126	46.7

**Fig. 2:**
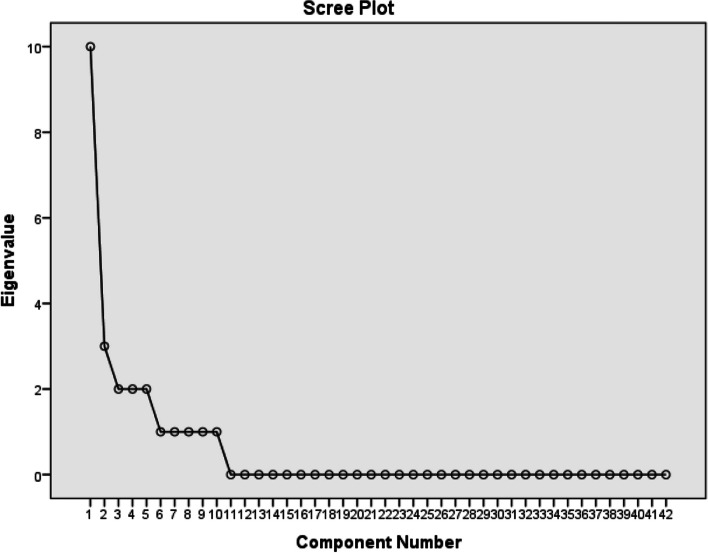
Scree plot of the exploratory factor analysis of EBET

Performing an EFA to uncover underlying variables, the KMO index was calculated, resulting in a value of 0.865. This suggests the sample’s suitability for analysis (χ2 = 5718.83, df = 861, *p* < .001). Additionally, the Bartlett Test of Sphericity was conducted to assess whether the correlation matrix resembles an identity matrix, indicating the appropriateness of the data for factor analysis. Factors were derived from the dataset using varimax rotation, a technique aimed at maximizing the variance of the squared loadings, and grouping highly correlated variables together. The scree plot revealed the presence of 10 factors, as illustrated in Fig. 2. Collectively, these factors accounted for 66.25% of the total variance in the data.

Subsequent to Varimax rotation and applying a factor loading threshold of 0.4, specific items associated with each factor were discerned [33]. This process necessitated the removal of 6 cases, while the remaining items exhibited a factor loading of at least 0.4 and were unequivocally assigned to a single factor. Notably, all items retained at this stage exhibited a commonality exceeding 0.5, with only two items falling slightly below this threshold but still maintaining a commonality above 0.4 [39].

..The majority of items demonstrated minimal issues with cross-loadings, indicating clear delineation between factors. However, items 17 and 18 exhibited cross-loadings with Factors 2 and 7, suggestive of a potential relationship between these factors. A threshold difference of 0.15 between factor loadings was considered acceptable for retaining items on the scale [40]. Given that the loadings of these items were notably stronger on one factor compared to others, and aligned with the theoretical framework, both items were retained and assigned to Factor F2, which demonstrated the strongest loading. Table 3 presents the factor loadings of the extracted factors along with the percentage of explained variance.

**Table 3:** Exploratory analysis of EBET questionnaire

**Component**	**Item**	F3	F1	F10	F7	F2	F8	F6	F5	F4	F9	**Communalities**
F3	1-I plan to stretch every day during rest for the next three months.	**0.803**	0.054	0.137	-0.093	0.021	0.013	0.151	0.215	0.055	0.181	0.783
	2-I have decided to maintain the correct body posture while working for the next three months.	**0.819**	0.133	0.217	-0.041	-0.100	-0.020	0.103	0.059	0.104	0.187	0.811
	3-I have decided to maintain the correct body posture for the next three months, even during period’s high pressure of at work.	**0.800**	0.115	0.210	0.111	-0.105	0.037	0.053	0.142	0.038	0.158	0.767
	4-I plan to continue stretching for the next three months while resting.	**0.799**	0.128	0.236	-0.082	-0.095	0.013	0.171	0.181	0.086	0.128	0.801
F1	5-The industrial hygienist helps me to stretch.	0.119	**0.696**	-0.070	0.164	-0.170	-0.095	0.075	0.114	0.038	0.013	0.641
	6-My colleagues remind me to observe the correct body posture during work.	0.065	**0.789**	0.206	-0.110	-0.118	-0.009	0.015	-0.104	0.112	0.144	0.731
	7-The supervisor of the workplace pays attention to the correct posture of my body during work.	0.059	**0.716**	0.156	0.046	-0.123	-0.102	0.041	-0.013	0.241	0.068	0.624
	8-During breaks, my colleagues invite me to do stretching exercises. In my work environment, I receive training about proper posture while working.	0.027	**0.738**	0.174	-0.141	-0.037	-0.044	0.163	0.122	0.086	0.114	0.631
	9-The industrial hygienist helps me to stretch.	0.154	**0.730**	0.071	-0.037	-0.080	-0.074	0.062	0.176	0.081	0.097	0.629
F10	10-I pay attention to the way my colleagues do stretching exercises.	0.226	0.240	**0.740**	-0.040	0.016	-0.019	0.043	0.156	0.193	0.089	0.751
	11-I repeat it by observing the correct body posture of my colleagues.	0.213	0.171	**0.788**	-0.028	0.030	0.084	0.014	0.060	0.135	0.100	0.757
	12- I pay attention to the correct body posture of my colleagues during work.	0.082	0.078	**0.829**	-0.027	0.021	-0.046	0.073	-0.007	0.117	0.173	0.752
	13-I practice it by watching how to do stretching movements.	0.334	0.212	**0.670**	-0.009	-0.078	0.012	0.077	0.150	0.080	0.154	0.684
F7	14-If I do stretch exercises while working, I will be approved by the supervisor of the workplace.	0.068	0.285	0.362	**0.450**	-0.059	-0.091	0.081	0.308	0.320	0.230	0.487
	15-If I follow the correct posture, the health expert will approve me.	0.174	0.309	0.355	**0.425**	-0.046	-0.070	0.149	0.354	0.372	0.113	0.561
	16-My family members encourage me to stretch.	0.287	0.070	0.321	**0.426**	0.166	-0.031	0.262	0.058	-0.062	0.265	0.504
F2	17-The high pressure of work prevents me from maintaining the correct posture of my body during work.	-0.009	-0.171	0.036	0.410	**0.755**	0.071	0.039	-0.102	-0.022	-0.083	0.637
	18-It is difficult for me to maintain the correct posture while doing my work.	-0.019	-0.088	-0.085	0.403	**0.714**	0.131	0.101	0.002	-0.055	-0.160	0.443
	19-Inappropriate chairs and equipment do not allow me to maintain the correct posture of my body during work.	0.131	0.006	0.007	0.257	**0.562**	-0.005	0.023	-0.062	0.121	0.117	0.577
	20-Observing the correct body posture during work slows down my work.	-0.208	-0.165	0.054	-0.012	**0.745**	0.081	-0.074	0.007	-0.044	-0.083	0.645
	21-Stretching during breaks robs me of the opportunity to rest.	-0.171	-0.060	0.013	0.367	**0.621**	0.072	-0.069	-0.007	-0.124	-0.075	0.577
	22-My colleagues make fun of me if I maintain the correct posture during work.	-0.004	-0.055	-0.025	0.099	**0.420**	-0.075	0.091	0.117	0.117	-0.041	0.611
	23-Due to the presence of cameras and other monitoring devices in the workplace, it is impossible to do stretching exercises.	-0.074	-0.025	-0.070	0.025	**0.414**	0.026	0.041	-0.018	-0.096	0.024	0.638
F8	24-Decreased musculoskeletal pain.	0.070	-0.042	-0.012	0.064	0.049	**0.856**	-0.009	-0.015	0.094	0.021	0.755
	25-Decreased fatigue.	0.007	-0.144	0.016	-0.076	0.094	**0.821**	0.117	0.021	-0.014	0.019	0.723
	26-Increasing my physical strength.	0.075	-0.044	0.027	0.092	0.059	**0.866**	0.028	-0.075	-0.046	0.066	0.784
	27-Postponing my physical disability.	-0.123	-0.046	-0.035	-0.112	0.090	**0.724**	0.150	0.087	-0.092	0.007	0.600
F6	28-Observing the correct body position during work reduces musculoskeletal pain.	0.218	0.019	0.040	0.041	-0.045	-0.013	**0.770**	-0.050	0.016	0.122	0.620
	29-Doing stretching exercises during breaks relieves my fatigue.	0.116	0.124	0.079	0.048	0.015	0.008	**0.743**	0.057	0.070	0.006	0.641
	30-Stretching improves my physical ability.	0.072	0.106	0.063	0.106	-0.020	0.132	**0.764**	0.085	0.207	0.120	0.699
	31-Observing the correct body position during work delays my physical disability.	-0.014	0.063	0.028	-0.080	0.089	0.193	**0.712**	0.119	-0.052	0.100	0.590
F5	32-It is easy for me to do stretching exercises correctly.	0.278	0.034	0.056	-0.138	-0.068	-0.020	0.060	**0.425**	0.220	0.559	0.676
	33-I have the ability to maintain the correct body position while working.	0.282	0.098	0.166	0.130	-0.236	0.092	0.122	**0.538**	0.100	0.392	0.593
	34-It is easy for me not to have long standing positions while working.	0.113	0.081	0.045	0.009	-0.060	-0.004	0.059	**0.780**	-0.031	0.207	0.735
	35-It is easy for me to do stretching exercises correctly.	0.343	0.109	0.192	0.067	0.006	0.027	0.079	**0.666**	0.039	0.099	0.647
F4	36-The hygienist expects me to stretch.	0.148	0.045	0.126	0.081	0.031	-0.031	0.147	0.025	**0.761**	0.077	0.655
	37-The head of the department confirms compliance with the correct body position.	0.042	0.210	0.087	0.062	-0.094	-0.007	0.005	-0.006	**0.782**	0.150	0.677
	38-Stretching in the workplace is accepted by the authorities.	-0.012	0.207	0.214	-0.163	-0.021	0.007	0.018	0.050	**0.714**	0.168	0.685
F9	39-I know how to do stretching exercises.	0.163	0.182	0.125	-0.227	0.052	0.022	0.071	0.182	0.166	**0.767**	0.700
	40-I know how to keep the right angles of my body while working.	0.163	0.137	0.099	-0.090	-0.028	0.059	0.128	0.179	0.242	**0.707**	0.702
	41-While working, I pay attention to maintaining and respecting the correct posture of my body.	0.175	0.152	0.285	0.211	-0.309	0.064	0.102	0.021	0.085	**0.620**	0.708
	42-I know how to do stretching exercises.	0.189	0.094	0.271	0.266	-0.205	0.052	0.195	0.085	0.010	**0.549**	0.695
**Variance Percentage**		24.63	8.95	6.19	5.40	4.85	4.24	3.56	3.05	2.84	2.50	
**Eigenvalues**		10.34	3.76	2.60	2.27	2.03	1.78	1.49	1.28	1.19	1.05	

*F1* Social Support, *F2* Barriers, *F3* Intention, *F4* Normative beliefs, *F5* Self-efficacy, *F6* Outcome expectations, *F7* Reinforcement, *F8* outcome expectancies, *F9* Behavioral skills, *F10* Observational learning

### Confirmatory factor analysis

**Fig. 3:**
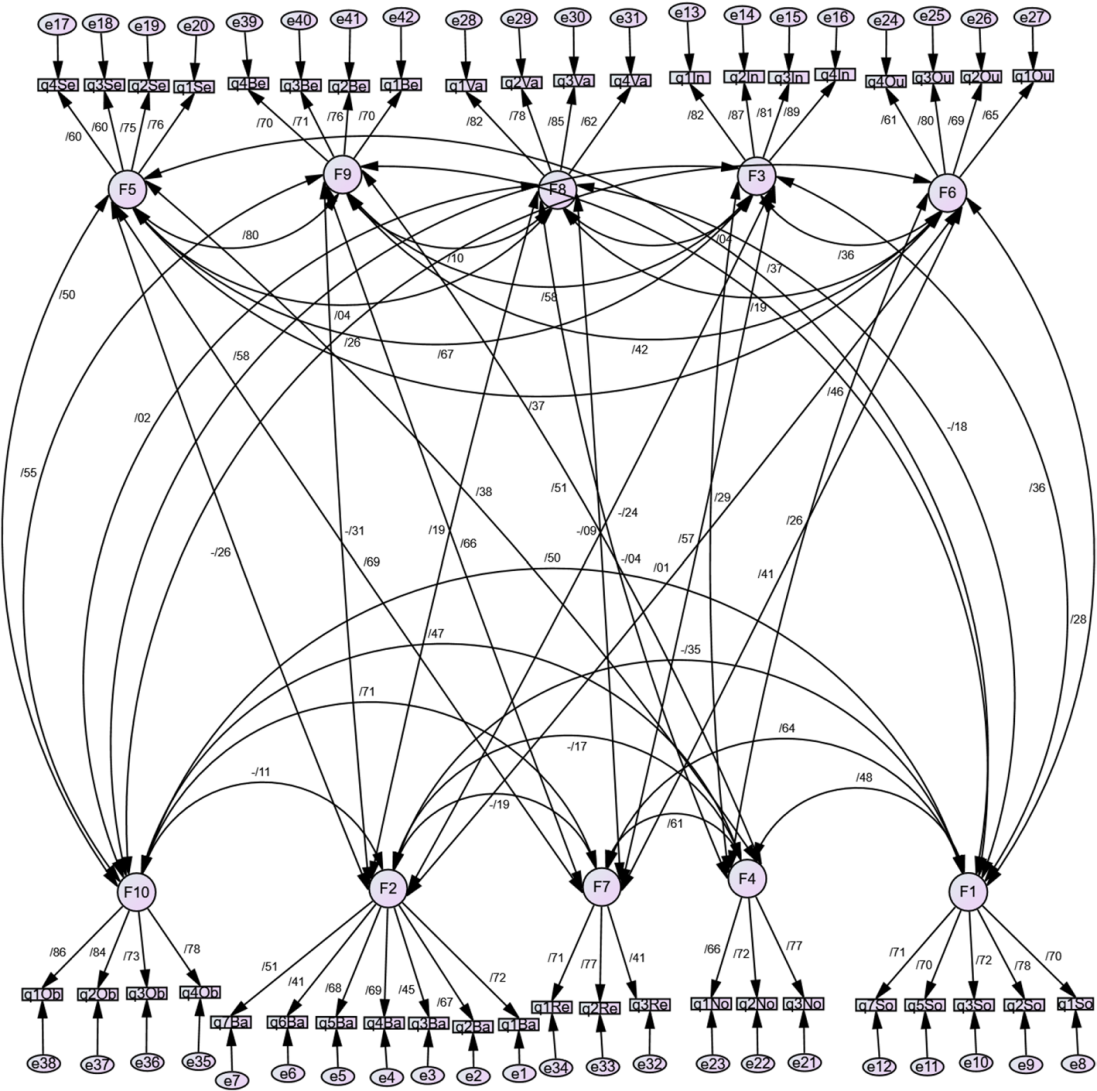
Confirmatory factor analysis model of EBET questionnaire

CFA was used to validate the structure obtained by EFA. Adequacy of model fit was evaluated by examining several indices including chi-square statistic, chi-square ratio, and degrees of freedom. The findings showed that the model showed good fit, as evidenced by the RMSEA of 0.051 and the chi-square ratio to degrees of freedom of 1.72 (Table 4). In conducting CFA, it is recommended that the absolute values of loadings should ideally exceed 0.3 to ensure optimal model performance [41]. In the present study, all items within each structural component demonstrated factor loadings surpassing 0.4, indicating robust associations between observed variables and underlying constructs (Fig. 3).

**Table 4:** Summary results of confirmatory factor analysis

χ2	χ2/df	p	CFI	TLI	RMSEA
1305.07	1.68	< 0.001	0.91	0.92	0.052

*χ2* chi-square, *χ2/df* normed chi-square, *CFI* comparative fit index, *TLI* Tucker, and Levix Index, *RMSEA* root mean square error approximation

### Reliability

The McDonald’s Omega coefficient was calculated as 0.740 for the entire scale, indicating acceptable internal consistency reliability [38]. Across various factors related to behavior change, median McDonald’s Omega coefficients ranged from 0.70 to 0.91, suggesting generally reliable measures within each factor. Interquartile ranges (IQRs) for these coefficients varied from 2 to 7, indicating variability in response dispersion (Table 5).

**Table 5:** The descriptive statistics and McDonald’s Omega values

Factors	McDonald’s Omega	Median	IQR
Outcome expectations	0.78	16	3
outcome expectancies	0.83	20	2
Normative beliefs	0.76	10	3
Barriers	0.78	26	7
Social support	0.84	8	5
Observational learning	0.87	12	4
Reinforcement	0.70	10	2
Behavioral skills	0.81	14	4
Self-efficacy	0.78	13	4
Intentions	0.91	13	4

*IQR* interquartile range

Once the validity and reliability of the EBET questionnaire were confirmed, it underwent the finalization process and was completed.

## Discussion

The utilization of psychometric evaluations is essential in guaranteeing dependable and valid outcomes when evaluating health-related behaviors. This approach facilitates the acquisition of significant data that can be utilized to make informed decisions [42]. Therefore, in order to improve ergonomic behaviors, it is essential to measure using standard and reliable tools. Moreover, WwAL are particularly vulnerable to MSDs [6]. The examination of ergonomic behaviors is crucial for identifying necessary corrective actions, including training. In this study, a psychometric tool has been developed to measure ergonomic behaviors among Iranian workers in assembly lines. The tool, called EBET, is validated and reliable, taking into account cognitive, environmental, and individual factors associated with MSDs. Our questionnaire expands on SCT by encompassing all fundamental constructs [27], In contrast to previous questionnaires on similar subjects that focused on a narrower range of constructs, this study encompassed a broader set of constructs [33]. To our knowledge, this is the initial questionnaire in Iran to evaluate and measure ergonomic behaviors of WwAL based on SCT constructs.

To improve the clarity of the sentences, face validity was utilized, and both content analysis and factor analysis were conducted. The participation of 10 expert panels resulted in a CVI of 0.97 and a CVR of 0.92, which align with the acceptable values outlined in the Lawshe table. As a result, the reported findings for CVI and CVR are considered appropriate [43].

The McDonald’s Omega analysis indicated that all questions demonstrated a reliability of 0.7 in the optimal condition, indicating satisfactory internal consistency. This value shows that the questions on the questionnaire are related to one another, and thus are consistent [38].

EFA revealed that 10 factors collectively explained 66.25% of the variance. This indicates that these factors adequately capture a substantial portion of the variability present in the observed variables. Intention was found to be the most influential factor, reflecting an individual’s commitment to performing ergonomic behaviors. This finding is consistent with other studies and is theoretically supported [44, 45]. Research consistently demonstrates that intention plays a pivotal role in shaping an individual’s commitment to ergonomic behaviors. This intention is influenced by various factors, such as attitude, perceived behavioral control, social influence, and support from management [46]. The level of “social support” accounted for 8.95% of the variance. This implies that employees perceiving higher levels of social support are inclined to adopt ergonomic practices, such as employing proper lifting techniques, maintaining good posture, or taking regular breaks, thus reducing the risk of musculoskeletal injuries. Studies have shown that social support is effective in reducing MSDs in the workplace [30]. Both Villotti and Henry emphasized the significance of social support within distinct populations. Their respective studies revealed that social support can enhance work productivity and health outcomes [31, 32]. This underscores the necessity of acknowledging social support as a crucial factor in fostering and sustaining favorable ergonomic practices within industrial settings. The third area of EBET focused on ‘observational learning,’ with the items of this factor explaining 6.19% of the total variance. This suggests that women’s ability to comprehend, pay attention to, and replicate appropriate ergonomic techniques through observational learning is crucial for the adoption of such behaviors in industrial settings. Observational learning plays a pivotal role in comprehending and implementing ergonomic principles within real work environments [18]. The fourth domain, “reinforcement”, encompassed three items and accounted for 5.40% of the total variance. This factor examined how individuals receive positive feedback and rewards from their supervisors or colleagues when they engage in ergonomically correct actions. Reinforcement plays a crucial role in motivating workers to consistently practice good ergonomics and ensuring their protection [18]. This finding suggests that when individuals receive recognition and rewards for engaging in ergonomically appropriate behaviors, it can serve as a motivational factor, encouraging them to persist in these behaviors. The fifth domain is “Perceived barriers” with seven items explaining 4.85% of the total variance. Khandan highlighted the prevalence of non-ergonomic behaviors in the workplace attributed to various obstacles [47]. The literature underscores the significance of both objective and subjective evaluations of environmental ergonomic factors, advocating for a comprehensive approach to overcoming barriers [48]. The remaining constructs, including outcome expectancies, outcome expectations, self-efficacy, normative beliefs, and behavioral skills, had variances ranging from 4.24% to 2.50%. Despite these factors explaining a smaller proportion of the variance, it is important to consider that the psychometric measures in our study encompass various constructs, each potentially contributing to the overall variance to different degrees. Therefore, these constructs may still offer unique insights within the framework. They might capture specific and fundamental aspects of the phenomenon not sufficiently represented by other constructs in the model. Previous research underscores the significance of these constructs in influencing health-related behavior change [49].

The CFI results further validate the suitability of the model. Additionally, CFI values exceeding 0.9, RMSEA below 0.08, GFI above 0.9, and a χ2/df ratio close to 1 indicate a favorable model fit [28, 33]. The outcomes of the CFA demonstrate that all analyzed structures and factors have achieved an acceptable level of fit, affirming the adequacy of the measurement model in representing the underlying theoretical constructs and accurately assessing the desired variables.

### Limitations and future studies

While our study has provided valuable insights into the reliability of the instrument, future research could employ it to measure factors influencing ergonomic behaviors in diverse working populations and evaluate the effectiveness of educational interventions targeting ergonomic improvements. Additionally, exploring the validity and reliability of this questionnaire across different societal groups could establish it as a robust tool for assessing ergonomic behaviors based on SCT.

However, it is crucial to acknowledge certain limitations. Although our tool considers individual, cognitive, and environmental factors influencing ergonomic behavior, the inclusion of numerous dimensions and questions in the questionnaire poses a challenge. Future research should aim to develop a condensed version of the questionnaire.

The tool primarily focused on ergonomic behaviors such as stretching movements and monitoring body posture during work activities. It is recommended that future studies explore additional ergonomic dimensions to provide a more comprehensive assessment.

Specifically, our study did not conduct convergent and discriminant analysis to assess the relationships between variables and the distinctiveness of the constructs. Furthermore, both EFA and CFA were performed simultaneously on a single sample, ensuring data consistency but potentially constraining the generalizability of the findings. Subsequent research should contemplate employing distinct samples for exploratory and confirmatory analyses to enhance result generalizability.

While research has emphasized the importance of assessing the discriminatory power and difficulty level of assessment tools [50], this specific aspect was not addressed in the present psychometric study. It is advisable for future investigations to incorporate the evaluation of these indices. Additionally, subsequent studies should explore other psychometric characteristics of the tool to ensure a comprehensive understanding of its properties.

## Conclusions

EBET serves as a reliable and valid tool to assess ergonomic behaviors in workers, relying on SCT. Researchers can use EBET to collect data and implement appropriate training interventions aimed at increasing ergonomic behavior among WwAL. However, it is important to acknowledge that EBET may not capture all aspects of ergonomic behaviors. Therefore, future efforts should prioritize evaluating the applicability of EBET among different worker populations and considering other dimensions of ergonomics to ensure its broader applicability and effectiveness.

## Data Availability

The datasets produced and/or analyzed in the present study are not accessible to the public because they contain personal information that could compromise confidentiality. However, interested individuals may request access to these datasets from the corresponding author.

## Abbreviations

WwAL: Women workers on assembly lines
WMSDs: Work-related musculoskeletal disorders
MSDs: Musculoskeletal disorders
SCT: Social cognitive theory
EBET: Ergonomic Behaviors Evaluation Tool
CVA: Content validity assesses
CVR: Content validity ratio
CVI: Content validity index
EFA: Exploratory factor analysis
CFA: Confirmatory factor analysis
KMO: Kaiser–Meyer–Olkin
CFI: Comparative fit index
GFI: Goodness of fit index
NFI: Normed fit index
RMSEA: Regarding root mean square error of approximation
